# Population-Level Health Intervention and Primary Care Quality for Veterans

**DOI:** 10.1001/jamanetworkopen.2025.44378

**Published:** 2025-11-18

**Authors:** Chelle L. Wheat, Ashok Reddy, Sarah E. Shirley, Kristen E. Gray, Susan E. Stockdale, Karin M. Nelson, Edwin S. Wong

**Affiliations:** 1 Center for Veteran-Centered and Value-Driven Care, VA Puget Sound Health Care System, Seattle, Washington; 2 Department of Health Systems and Population Health, University of Washington, Seattle; 3 Division of General Internal Medicine, Department of Medicine, University of Washington, Seattle; 4 VA Greater Los Angeles Healthcare System, Center for the Study of Healthcare Innovation, Implementation and Policy, Los Angeles, California; 5 Division of General Internal Medicine, Department of Medicine, David Geffen School of Medicine, University of California, Los Angeles

## Abstract

**Importance:**

The COVID-19 pandemic caused substantial disruptions to primary care, potentially impacting quality of care for chronic conditions. The Veterans Health Administration (VHA) implemented the Preventive Health Inventory (PHI) initiative, a multicomponent care management intervention, focused on chronic disease care and preventive screening to support the delivery of delayed care.

**Objective:**

To examine the association of PHI implementation with measures of primary care quality and potentially avoidable health care use.

**Design, Setting, and Participants:**

This cohort study used VHA administrative data of multiple propensity score–matched cohorts of veterans with diabetes and/or hypertension enrolled in primary care who received or did not receive care via the PHI. Data were collected from February 1, 2021, through February 28, 2022, and analyzed between April 30 and June 28, 2024.

**Exposure:**

Templated electronic health record note documenting use of the PHI.

**Main Outcomes and Measures:**

A difference-in-differences approach was used to evaluate the association of PHI receipt with diabetes and hypertension clinical quality measures (eg, poor diabetes control with a hemoglobin A_1c_ >9% [75 mmol/mol]) and preventable use outcomes, including outpatient care use, preventable emergency department visits, and hospitalizations for ambulatory care–sensitive conditions.

**Results:**

For each outcome, separate cohorts of veterans who received the PHI intervention (from 8434 [statin therapy] to 97 695 [preventable care use outcomes]) were propensity score matched with those who did not (from 5574 [statin therapy] to 118 188 [preventable care use outcomes]). Veterans who received care using the PHI tool had a lower probability of poor diabetes control (2.9 percentage points [95% CI, −3.8 to −1.9 percentage points]) and a higher probability of adequate blood pressure control (4.0 percentage points [95% CI, 2.6-5.3 percentage points]). No differences were observed between groups for statin therapy or preventable use outcomes. Use of the PHI was associated with increased outpatient care use (310 [95% CI, 272-348] visits per 1000 veterans).

**Conclusions and Relevance:**

This cohort study found that implementation of VHA’s PHI was associated with improved care quality of care for diabetes and hypertension and did not increase preventable care use, suggesting that the PHI may serve as a model for population health approaches that seek to reengage inactive patients since the COVID-19 pandemic.

## Key Points

**Question:**

What is the association of the Preventive Health Inventory (PHI), a multicomponent care management tool, with primary care quality measures for diabetes, hypertension, and preventable health care use?

**Findings:**

In this cohort study of multiple propensity score–matched cohorts of veterans with diabetes and/or hypertension, those who received care using the PHI had improved diabetes and hypertension control and no associated increase in preventable health care use compared with similar veterans not receiving PHI.

**Meaning:**

These findings suggest that the use of a multicomponent care management intervention may serve as a model for population health approaches that seek to reengage inactive patients since the COVID-19 pandemic.

## Teaser

This cohort study examines the association of the Veterans Health Administration’s Preventive Health Inventory implementation with measures of primary care quality and preventable health care use.

## Introduction

Diabetes and hypertension are 2 commonly managed conditions in primary care and are especially prevalent in the veteran population. One-quarter of the veteran population has diabetes, and 71% have a blood pressure of at least 140/90 mm Hg, with even greater numbers classified as hypertensive if using updated guidelines.^1,2,3^ During the COVID-19 pandemic, there was longer follow-up between blood pressure measurements, which likely contributed to an increase in the number of veterans not achieving appropriate blood pressure control.^4^ Similarly, there were notable disruptions to care for veterans with diabetes during the pandemic, including reductions in hemoglobin A_1c_ (HbA_1c_) measurements, prescription fills for diabetes medications, and follow-up visits.^5^ Considering the importance of routine monitoring and follow-up of individuals with diabetes and/or hypertension, this and prior evaluations of COVID-19 impacts on care have focused on patients with these conditions.

In response to the declines in primary care services during the COVID-19 pandemic,^6,7,8^ the Veterans Health Administration (VHA) Office of Primary Care developed a population-level, multicomponent care management intervention designed to catch up patients on care that was delayed or disrupted.^9^ The Preventive Health Inventory (PHI) leverages the VHA’s use of the patient-centered medical home model, which promotes team-based care, including primary care nurses. The PHI intervention includes a national dashboard of quality measures, a telehealth visit with a primary care nurse manager, and a templated electronic health record (EHR) note with a checklist of care needs that the nurse completes. The templated note covers chronic disease management, cancer prevention activities, and screening and management of mental health concerns (eTable 1 in Supplement 1). Although the VHA has existing, routinely monitored quality measures in primary care, the PHI is unique in that its components are used to streamline and prioritize care, focusing on specific aspects of primary care and their associated quality measures.

There is limited research on the effectiveness of population-based interventions designed to catch up on care disrupted by the pandemic. Two prior evaluations of the PHI focused on clinic-level outcomes of adoption for these diabetes and hypertension care measures. The first showed that primary care clinics in the highest 10% of use for the PHI had improved quality-of-care metrics, including diabetes control (HbA_1c_ <9% [75 mmol/mol]), diabetes screening, and blood pressure control (<140/90 mm Hg).^9^ The second suggested that the PHI intervention was equitably administered across racial and ethnic groups, an especially important finding given the existing inequities in these groups’ diabetes and hypertension burden and management.^10^ The focus of the current evaluation was to estimate the outcomes of the PHI intervention associated with care quality and to compare veterans who received care via the PHI with those who did not. We conducted a retrospective study of VHA administrative data with 2 main objectives: (1) to examine key primary care quality outcomes and their association with use of the PHI intervention and (2) to explore potentially avoidable use measures, such as preventable emergency department (ED) visits and hospitalizations for ambulatory care–sensitive conditions (ACSCs) and their association with use of the PHI.

## Methods

### Overview

This retrospective cohort study examined data of veterans who received care via the PHI between February 1, 2021, when the intervention was started nationally, and February 28, 2022. We compared this cohort with a matched comparison group of veterans who did not receive care via the PHI and examined the PHI’s association with measures of primary care quality and health care use. The study was approved by the US Department of Veterans Affairs Central Institutional Review Board with a waiver of informed consent as the research was considered to have no more than minimal risk to participants. This study followed the Strengthening the Reporting of Observational Studies in Epidemiology (STROBE) reporting guideline for cohort studies.

### Data Sources

The data for this study were obtained from VHA’s Corporate Data Warehouse, a national repository of clinical and administrative data from the VHA’s EHR system. Corporate Data Warehouse data include information on all health care encounters provided by the VHA, notably diagnosis codes, service procedure codes, and screening measures. For this study, we extracted a specific set of clinical reminders created to track the implementation and use of the PHI. Clinical reminders prompt primary care teams for needed preventive care and provide a template for recording completion. We extracted national electronic quality measure (eQM) data, similar to Healthcare Effectiveness Data and Information Set data,^11^ which capture several measures of diabetes and hypertension care quality. Electronic quality measure data use applied algorithms that automate calculation of care quality in administrative data and a point-in-time calculation for the full VHA-enrolled population.^12^ The eQM data were extracted from February 1, 2019, through February 28, 2022, to observe trends both before and after PHI implementation.

### Study Sample

Our cohorts included veterans who were assigned a primary care practitioner and alive as of February 28, 2022. Separate cohorts were created for diabetes, hypertension, and preventable health care use. Cohorts were examined separately because each outcome had distinct inclusion and exclusion criteria and covered different chronic conditions. For the quality measures, veterans also were required to have a confirmed diagnosis of hypertension and/or diabetes. For diabetes, a confirmed diagnosis was defined as at least 2 diagnoses of diabetes per *International Classification of Diseases, Ninth Revision* or *International Statistical Classification of Diseases, Tenth Revision* codes on 2 separate dates or, alternatively, dispensation of insulin or hypoglycemia or antihyperglycemia medications along with a diagnosis code for diabetes. For hypertension, a confirmed diagnosis was at least 2 separate dates on which an *International Classification of Diseases, Ninth Revision* or *International Statistical Classification of Diseases, Tenth Revision* diagnosis code for hypertension was recorded. The look-back period for each measure was 12 months, and each measure was evaluated monthly. For the preventable use and sensitivity analysis of outpatient care use, the cohort was not limited to veterans with a diagnosis of diabetes and/or hypertension and included the entire primary care population. We then examined whether veterans had received care via the PHI during our study period, defined as the presence of a completed clinical reminder, and created an indicator for PHI receipt. If a veteran had multiple records of receiving the PHI, the earliest date of record was used. Veterans who did not have observable data for the entire study period were excluded.

Propensity score–matched cohorts of veterans who received care via the PHI and their comparators who did not receive care via the PHI were created by using 2 controls: 1 PHI match with replacement using nearest neighbor matching and 1 applying a caliper of 0.2 SDs of the propensity score for each outcome.^13^ We used matching with replacement to reduce bias and ensure high-quality matches. A prespecified standardized mean difference (SMD) of 0.1 was used to determine match quality.^14^ We selected variables for propensity score matching based on their potential association with the exposure (PHI) and quality outcomes. Veterans were matched on age (years); sex (male or female); marital status (married or other [single, separated or divorced, widowed]); self-reported race and ethnicity (American Indian or Alaska Native; Asian, Native Hawaiian, or Pacific Islander; Black; Hispanic; White)^15^; priority status (1-3, 4-6, or 7-8), which is an indirect proxy for socioeconomic status that considers service-connected disabilities^16^; rurality (urban, rural, or highly rural or insular islands); drive distance to primary care (per 10 miles); outpatient use in the prior year; Care Assessment Need score^17^; Gagne comorbidity score^18^; month and year of PHI receipt or of the most recent primary care visit for those with no PHI receipt; socioeconomic index decile (based on census data), which is a surrogate marker for income^19^; primary care practitioner’s VHA tenure (years); primary care clinic’s overall PHI use; primary care clinic’s staffing ratio (number of support staff for each practitioner); and primary care practitioner’s panel fullness (number of primary care patients adjusted for full-time equivalents, which is the number of hours an employee works in a standard pay period, with 1 considered full time). Covariates were drawn from administrative databases (including geographic data based on Rural-Urban Commuting Area codes), and the value from the quarter prior to the date of last PHI observation (February 28, 2022) was selected unless otherwise specified.

### Outcome Measures

eTable 2 in Supplement 1 outlines the primary care quality measures used in the study. The numerators were the number of veterans who met the individual criteria for that specific measure. For example, for the proportion of patients with diabetes with poor HbA_1c_ control, the denominator was all veterans aged 18 to 75 years with a documented diagnosis of diabetes. The numerator was the number of these veterans whose HbA_1c_ values were greater than 9% (75 mmol/mol) or who had no evidence of having their HbA_1c_ measured in the prior year.

Potentially avoidable heath care use outcomes are also presented in eTable 2 in Supplement 1. Preventable ED visits were those that could have occurred in a nonemergency setting, such as the primary care clinic with adequate care management. These visits were identified using the New York University Emergency Department Algorithm, which uses diagnosis codes to assign probabilities that an ED visit belongs in 1 of the following 4 categories: nonemergent; emergent, but primary care treatable; emergent ED preventable or avoidable; and emergent ED care needed.^20,21^ We calculated the expected number of preventable ED visits by summing nonemergent and emergent, but primary care treatable probabilities across all classified ED visits for a given patient.^22^ Hospitalizations for ACSCs were constructed using the Agency for Healthcare Research and Quality’s Prevention Quality Indicators at the patient level.^23,24,25^ We used the count of all VHA outpatient care use over our study period as a sensitivity outcome measure. The time frame for all analyses was February 1, 2019, to February 28, 2022.

### Statistical Analysis

The data for this study were analyzed between April 30 and June 28, 2024. We used a difference-in-differences (DID) regression model to separately evaluate the association of PHI receipt with each outcome of interest, using each propensity score–matched cohort of veterans who received care via the PHI and veterans who did not receive care via the PHI. We then implemented the Puhani DID estimator^26^ to estimate the before-and-after change in outcomes, and included a fixed effect that captured veterans assigned a primary care practitioner. We adjusted models for any covariates that had residual imbalances after matching. We used logistic regression for binary outcomes (eQMs) and Poisson models for count outcomes. We calculated SEs using the delta method and clustered by veteran. We assessed the parallel trends assumption by visual inspection of graphical trends of each outcome, as well as by estimating regressions that included the covariates, a linear time trend, group assignment (PHI receipt or nonreceipt), and the interaction between time trend and group assignment. Although there is no direct way to test whether the parallel trends assumption holds, visual inspection of trend graphs and no evidence of an interaction between group assignment and time in the regression model provided evidence in support of this assumption. All statistical analyses were performed using R, version 4.4.1 (R Foundation for Statistical Computing) and the following packages: MatchIt,^27^ marginaleffects,^28^ and survey.^29^ A threshold of *P* < .05 was used to assess statistical significance.

## Results

### Propensity Score–Matched Cohorts

**Figure 1.**
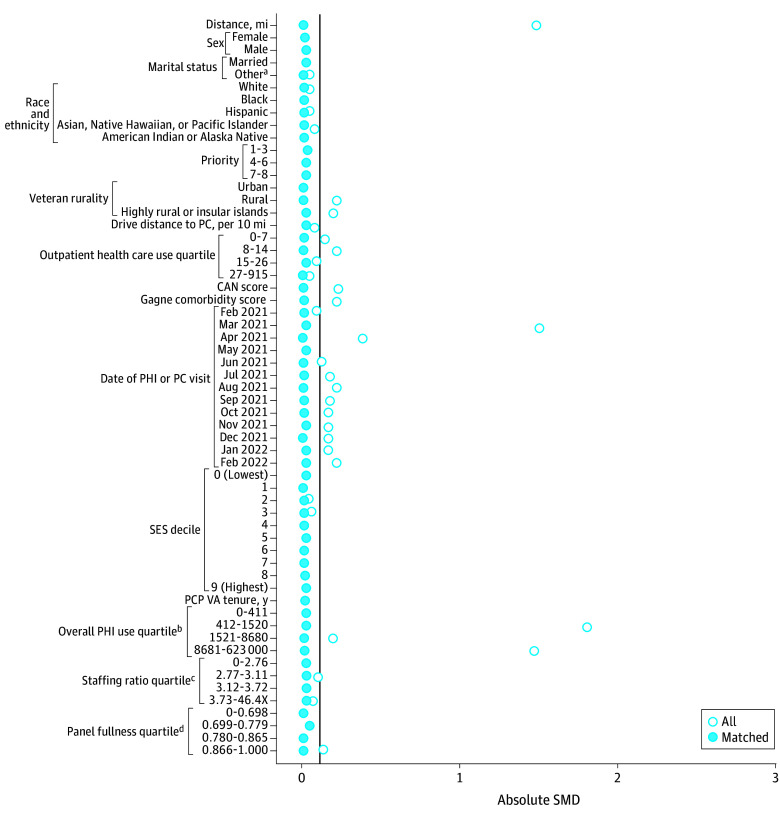
Standardized Mean Difference (SMD) Plot of Match Quality After Propensity Score Matching for Poor Diabetes Control Vertical line indicates SMD of 0.1. CAN indicates Care Assessment Need; PC, primary care; PCP, primary care practitioner; PHI, Preventive Health Inventory; SES, socioeconomic status; VA, US Department of Veterans Affairs. ^a^Other marital status included single, separated or divorced, or widowed. ^b^Number of patients. ^c^Number of support staff per practitioner. ^d^Number of primary care patients adjusted for full-time equivalents.

**Figure 2.**
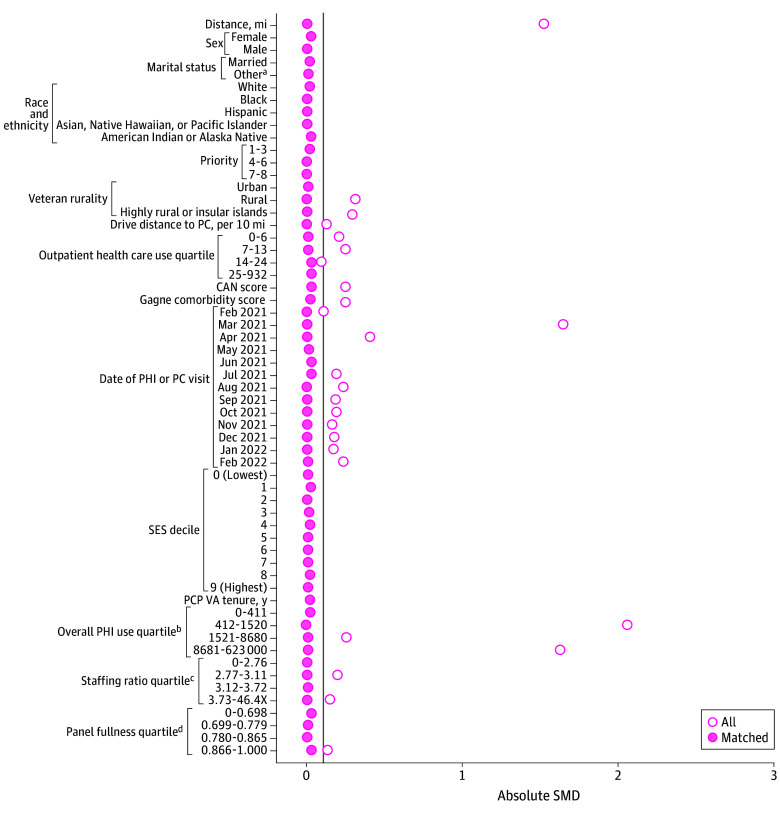
Standardized Mean Difference (SMD) Plot of Match Quality After Propensity Score Matching for Controlling High Blood Pressure Vertical line indicates SMD of 0.1. CAN indicates Care Assessment Need; PC, primary care; PCP, primary care practitioner; PHI, Preventive Health Inventory; SES, socioeconomic status; VA, US Department of Veterans Affairs. ^a^Other marital status included single, separated or divorced, or widowed. ^b^Number of patients. ^c^Number of support staff per practitioner. ^d^Number of primary care patients adjusted for full-time equivalents.

**Figure 3.**
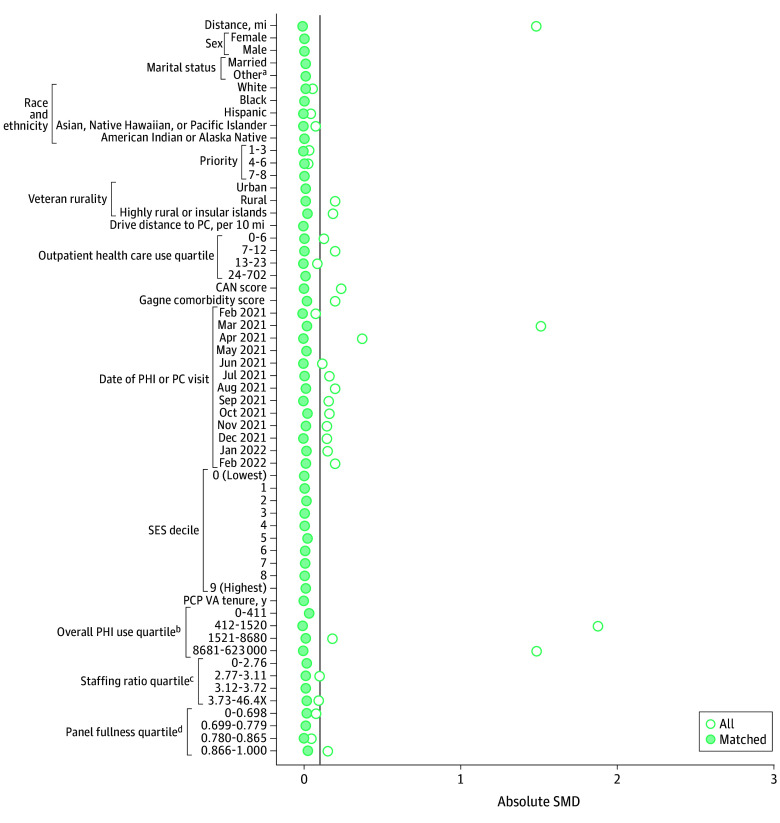
Standardized Mean Difference (SMD) Plot of Match Quality After Propensity Score Matching for Statin Therapy Among Patients With Diabetes Vertical line indicates SMD of 0.1. CAN indicates Care Assessment Need; PC, primary care; PCP, primary care practitioner; PHI, Preventive Health Inventory; SES, socioeconomic status; VA, US Department of Veterans Affairs. ^a^Other marital status included single, separated or divorced, or widowed. ^b^Number of patients. ^c^Number of support staff per practitioner. ^d^Number of primary care patients adjusted for full-time equivalents.

**Figure 4.**
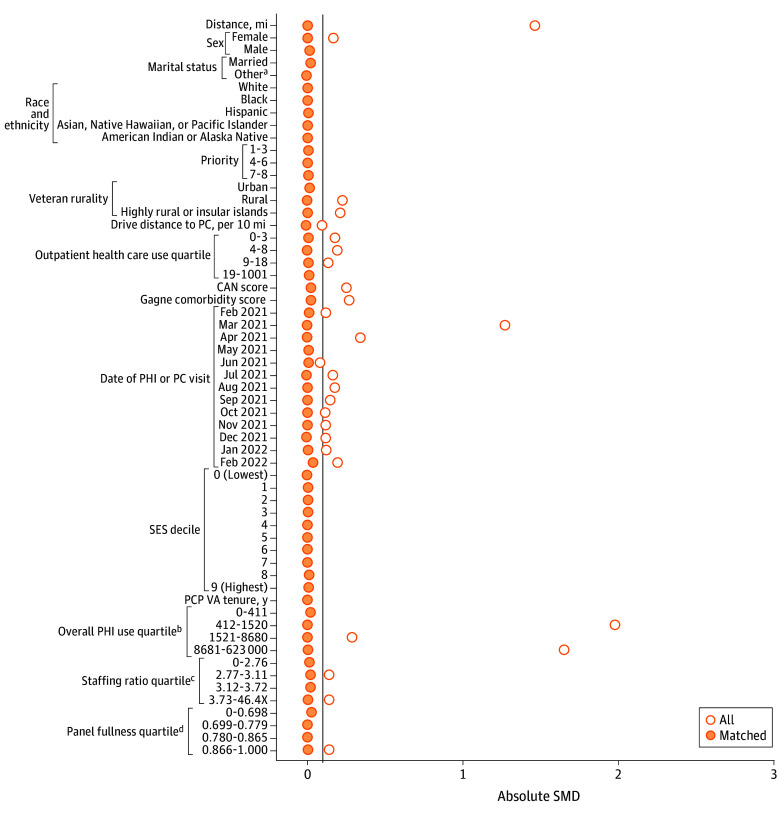
Standardized Mean Difference (SMD) Plot of Match Quality After Propensity Score Matching for Preventive Health Care Use Outcomes Vertical line indicates SMD of 0.1. CAN indicates Care Assessment Need; PC, primary care; PCP, primary care practitioner; PHI, Preventive Health Inventory; SES, socioeconomic status; VA, US Department of Veterans Affairs. ^a^Other marital status included single, separated or divorced, or widowed. ^b^Number of patients. ^c^Number of support staff per practitioner. ^d^Number of primary care patients adjusted for full-time equivalents.

The Table summarizes the samples for each propensity score–matched cohort. Sample sizes for control groups (ie, not receiving PHI) ranged from 5574 veterans (statin therapy) to 118 188 veterans (use outcomes; ie, preventable ED visits, hospitalizations for ACSCs, outpatient use). Exposed groups (receiving PHI) ranged from 8434 veterans (statin therapy) to 97 695 (use outcomes). Descriptive statistics for each cohort before and after matching are provided in eTables 3 to 6 in Supplement 1. Overall, cohorts mainly included male and non-Hispanic White veterans living in urban areas. Although we specified the matches to be 2 control veterans for every 1 veteran receiving PHI, we specified a caliper of 0.2 SD of the propensity score to ensure a high-quality match; therefore, in some cases, there were not 2 control veterans for each exposed veteran. The SMDs overall and for each variable included before and after matching are shown in Figure 1 for veterans with poor diabetes control, Figure 2 for veterans with high blood pressure, Figure 3 for veterans receiving statin therapy, and Figure 4 for use outcomes. The SMDs before and after propensity score matching for each variable are detailed in eTables 3 through 6 in Supplement 1. For each cohort, all final SMDs were less than the prespecified threshold of 0.1, indicating good quality matches overall.^30^

**Table.** Percentage Differences in Outcomes Between Veterans Who Received (Treated) and Did Not Receive (Control) the PHI Intervention

Outcome and propensity score–matched cohorts	Sample size	Difference between PHI recipients and nonrecipients (95% CI)
**Primary care quality measure** ^a^		
HbA_1c_ poor control		
Control	7538	−2.9 (−3.8 to −1.9)
Treated	11 468	
Statin therapy for patients with diabetes		
Control	5574	0.08 (−0.05 to 1.63)
Treated	8434	
High blood pressure control		
Control	10 738	4.0 (2.6 to 5.3)
Treated	17 433	
**Preventable health care use** ^b^		
Preventable ED visits		
Control	118 188	9.9 (−3.9 to 20.1)
Treated	97 695	
Hospitalizations for ACSCs		
Control	118 188	4.4 (−5.3 to 14.1)
Treated	97 695	
Outpatient care use		
Control	118 188	310 (272 to 348)
Treated	97 695	

Abbreviations: ACSC, ambulatory care–sensitive condition; ED, emergency department; HbA_1c_, hemoglobin A_1c_; PHI, Preventive Health Inventory.

^a^ Differences reported as percentage points.

^b^ Differences reported as visits per 1000 veterans.

### Unadjusted Trends in Outcomes

The unadjusted trends for each outcome are displayed in eFigures 1 to 6 in Supplement 1. The eQM outcomes are reported as proportions and use outcomes as counts. All eQM outcome trends before and after PHI implementation were similar between veterans receiving and not receiving PHI. Regression results for each eQM outcome showed additional evidence that the parallel trends assumption held for these outcomes. Although trends for PHI recipients and PHI nonrecipients showed some differences, the regression results showed no statistically significant departures from the parallel trends assumption.

### DID Models

Use of PHI was associated with a lower probability of poor diabetes control. Specifically, the before-and-after change in the probability of not achieving HbA_1c_ control was 2.9 percentage points lower (95% CI, −3.8 to −1.9 percentage points) among PHI recipients compared with nonrecipients. For statin therapy for veterans with diabetes, PHI recipients and nonrecipients had similar rates (0.08% [95% CI, −0.05% to 1.63%]). The before-and-after change in the probability of controlling blood pressure among veterans with hypertension was 4.0 percentage points (95% CI, 2.6-5.3 percentage points) higher for PHI recipients compared with nonrecipients. For both main preventable use outcomes, ie, hospitalizations for ACSCs and preventable ED visits, PHI recipients and nonrecipients had similar rates (9.9 [95% CI, −3.9 to 20.1] visits per 1000 veterans and 4.4 [95% CI, −5.3 to 14.1] visits per 1000 veterans, respectively). The sensitivity analysis of all VHA outpatient care use showed that PHI recipients had 310 visits per 1000 veterans (95% CI, 272-348 visits per 1000 veterans) more than nonrecipients (Table).

## Discussion

This cohort study of the VHA’s national implementation of the PHI found that the PHI increased control of diabetes and hypertension on several key care quality indicators. The outcomes for patients with diabetes and hypertension were clinically significant given the prevalence of these 2 conditions among US veterans. It is estimated that approximately 25% of veterans have diabetes and between 71% and 87% have hypertension,^1,2,3^ meaning that given the more than 9.2 million veterans enrolled in VHA,^31^ approximately 2.3 million have diabetes and 8.0 million have hypertension. Thus, the potential impact of the PHI intervention if applied to all veterans is an estimated 69 000 fewer veterans with poor diabetes control and 320 000 more veterans with controlled blood pressure.

Our findings contribute to the limited evidence on the outcomes associated with care management approaches, such as the PHI, embedded within the patient-centered medical home, a care delivery model specifically designed to improve care coordination.^32,33^ Evidence has suggested that care management could be especially beneficial for individuals living with multiple comorbid conditions and those at high risk for health inequities who frequently have both diabetes and hypertension.^34,35^ For these reasons, care management is especially important in the VHA population as veterans have an average of 5 chronic conditions.^36,37,38^ Veterans also often have socioeconomic disadvantages that place them at high risk for experiencing health care disparities.^39,40^ Many of these challenges were compounded during and following the COVID-19 pandemic when health care services were disrupted or delayed.^41,42,43^

Hospitalizations for ACSCs and preventable ED visits are traditionally used to identify preventable health care use. Importantly, our study did not find an association with preventable health care use. Gaps in care coordination are associated with an increased risk of preventable use, such as ED visits and hospitalizations, including associated increases in costs.^44,45^ The PHI aims to facilitate care coordination and, like other care coordination interventions, may be successful in preventing unnecessary health care use, especially among individuals experiencing delayed care.^46,47,48,49^ In addition to possibly preventing inappropriate use, the PHI was positively associated with the number of overall outpatient visits. This use may be influenced by efforts to reengage with primary care and other VHA services given the care disruptions caused by the pandemic.^50,51,52,53^ This finding is similar to that of other interventions involving outreach following care disruptions not limited to the pandemic.^51,54,55,56,57^ The PHI could serve as a model for other reengagement interventions outside pandemic recovery efforts.

These results complement prior findings regarding the PHI.^9,10,58^ From a clinic-level analysis, clinics that use the PHI the most have better quality outcomes in diabetes and blood pressure control than those that use the PHI to a lesser degree (ie, those in the bottom 10% of use). Furthermore, more outpatient use and higher Care Assessment Needs scores are associated with PHI receipt. Finally, PHI is being equitably administered across racial and ethnic groups. Future research should focus on barriers and facilitators to PHI use. Given the many benefits associated with the PHI and its potential for future use to engage veterans with primary care, it is vital for decision makers to understand which strategies are successful in promoting widespread adoption and sustainability of the PHI and related EHR-based care coordination tools.

### Limitations

This study had several limitations. First, we assessed VHA electronic use of templates as a proxy rather than as specific activities. However, this approach is policy salient and urgently needed given that VHA has made major nationwide investments in the use of templates. Second, though large and national, our administrative datasets may lack relevant clinical information, including care that is completed outside the VHA or by the small number of sites that have adopted a new EHR system. Third, the consensus outcome measures analyzed in this study do not capture all aspects of quality and use; however, these measures are both standard and widely used, including outside the VHA. Fourth, given the observational design, findings may be subject to confounding. However, we used a series of measures, including multivariable adjustment using a number of patient, practitioner, and clinic characteristics; propensity score matching; and use of fixed effects in both propensity score matching and DID models, to mitigate confounding. Fifth, the PHI initiative was not universally adopted across VHA clinics; thus, there was variability in its implementation, which could have biased our results. To mitigate the influence of this potential variability, we matched at the veteran level. In addition, we matched on characteristics thought to address selection bias given the voluntary nature of PHI receipt. Finally, results may not generalize to all patients, especially those who were at high risk of mortality. The PHI was rolled out primarily to capture data on veterans for whom chronic and preventive care was of primary salience. Quality measures were not applicable or appropriate for patients with a high risk of mortality in 1 year. However, receipt of chronic and preventive care through VHA is important to a majority of veterans.^59^

## Conclusions

In this cohort study, the PHI intervention, a national care management approach implemented by the VHA Office of Primary Care to improve care coordination, was associated with increases in diabetes and hypertension care quality and not with inappropriate or unnecessary care. Furthermore, the PHI was associated with increases in outpatient care use among veterans who had care disrupted by the pandemic. These findings have important implications. First, they suggest that relatively low workforce burden and telehealth interventions could have dramatic positive outcomes for veterans’ health at the population level. Second, telehealth interventions could serve as a touchpoint for veterans to receive necessary follow-up care, especially those with chronic conditions. Finally, this study shows that interventions such as the PHI are population-level health interventions that could be used for engaging veterans in primary care regardless of the reason for care disruption.

## Notes

Supplement 1. **eTable 1.** Description of PHI Services **eTable 2.** Chronic Disease Management Measures (eQMs) and Potentially Preventable Utilization Measures **eTable 3.** Propensity Match Variable Pre and Post Match, HbA_1c_ Poor Control **eTable 4.** Propensity Match Variable Pre and Post Match, Statin Therapy for Patients With Diabetes **eTable 5.** Propensity Match Variable Pre and Post Match, Controlling High Blood Pressure **eTable 6.** Propensity Match Variable Pre and Post Match, Utilization Outcomes (Preventable ED Visits, Ambulatory Care Sensitive Hospitalizations, Outpatient VA Utilization) **eFigure 1.** Monthly eQM Trends of Diabetes Control Pre and Post PHI Introduction: PHI Users vs Non–PHI Users **eFigure 2.** Monthly eQM Trends of Statin Use in Diabetes Pre and Post PHI Introduction: PHI Users vs Non–PHI Users **eFigure 3.** Monthly eQM Trends of Blood Pressure Control for Hypertension Pre and Post PHI Introduction: PHI Users vs Non–PHI Users **eFigure 4.** Monthly eQM Trends of Ambulatory Care Sensitive Condition Hospitalizations Pre and Post PHI Introduction: PHI Users vs Non–PHI Users **eFigure 5.** Monthly eQM Trends of Preventable Emergency Department Visits Pre and Post PHI Introduction: PHI Users vs Non–PHI Users **eFigure 6.** Monthly eQM Trends of VA Outpatient Utilization Pre and Post PHI Introduction: PHI Users vs Non–PHI Users

Supplement 2.
Data Sharing Statement
