# Supplementary material for: Population-Level Health Intervention and Primary Care Quality for Veterans
Source: JAMA Netw Open. 2025 Nov 18;8(11):e2544378. doi: 10.1001/jamanetworkopen.2025.44378 (PMC12628105; doi:10.1001/jamanetworkopen.2025.44378)
Supplement: Supplement 1. — eTable 1. Description of PHI Services eTable 2. Chronic Disease Management Measures (eQMs) and Potentially Preventable Utilization Measures eTable 3. Propensity Match Variable Pre and Post Match, HbA1c Poor Control eTable 4. Propensity Match Variable Pre and Post Match, Statin Therapy for Patients With Diabetes eTable 5. Propensity Match Variable Pre and Post Match, Controlling High Blood Pressure eTable 6. Propensity Match Variable Pre and Post Match, Utilization Outcomes (Preventable ED Visits, Ambulatory Care Sensitive Hospitalizations, Outpatient VA Utilization) eFigure 1. Monthly eQM Trends of Diabetes Control Pre and Post PHI Introduction: PHI Users vs Non–PHI Users eFigure 2. Monthly eQM Trends of Statin Use in Diabetes Pre and Post PHI Introduction: PHI Users vs Non–PHI Users eFigure 3. Monthly eQM Trends of Blood Pressure Control for Hypertension Pre and Post PHI Introduction: PHI Users vs Non–PHI Users eFigure 4. Monthly eQM Trends of Ambulatory Care Sensitive Condition Hospitalizations Pre and Post PHI Introduction: PHI Users vs Non–PHI Users eFigure 5. Monthly eQM Trends of Preventable Emergency Department Visits Pre and Post PHI Introduction: PHI Users vs Non–PHI Users eFigure 6. Monthly eQM Trends of VA Outpatient Utilization Pre and Post PHI Introduction: PHI Users vs Non–PHI Users [file jamanetwopen-e2544378-s001.pdf]

Supplemental Online Content

Wheat CL, Reddy A, Shirley SE, et al. Population-level health intervention and primary care quality for veterans. *JAMA Netw Open*. 2025;8(11):e2544378.
doi:10.1001/jamanetworkopen.2025.44378

eTable 1. Description of PHI Services

eTable 2. Chronic Disease Management Measures (eQMs) and Potentially Preventable Utilization Measures

eTable 3. Propensity Match Variable Pre and Post Match, HbA_{1c} Poor Control

eTable 4. Propensity Match Variable Pre and Post Match, Statin Therapy for Patients With Diabetes

eTable 5. Propensity Match Variable Pre and Post Match, Controlling High Blood Pressure

eTable 6. Propensity Match Variable Pre and Post Match, Utilization Outcomes (Preventable ED Visits, Ambulatory Care Sensitive Hospitalizations, Outpatient VA Utilization)

eFigure 1. Monthly eQM Trends of Diabetes Control Pre and Post PHI Introduction: PHI Users vs Non–PHI Users

eFigure 2. Monthly eQM Trends of Statin Use in Diabetes Pre and Post PHI Introduction: PHI Users vs Non–PHI Users

eFigure 3. Monthly eQM Trends of Blood Pressure Control for Hypertension Pre and Post PHI Introduction: PHI Users vs Non–PHI Users

eFigure 4. Monthly eQM Trends of Ambulatory Care Sensitive Condition Hospitalizations Pre and Post PHI Introduction: PHI Users vs Non–PHI Users

eFigure 5. Monthly eQM Trends of Preventable Emergency Department Visits Pre and Post PHI Introduction: PHI Users vs Non–PHI Users

eFigure 6. Monthly eQM Trends of VA Outpatient Utilization Pre and Post PHI Introduction: PHI Users vs Non–PHI Users

This supplemental material has been provided by the authors to give readers additional information about their work.

© 2025 Wheat CL et al. *JAMA Network Open*.

eTable 1. Description of PHI Services

Service Type	Preventive Screening	Chronic Condition Monitoring
**Counseling**	• Blood pressure<br>• Influenza vaccine<br>• Colorectal cancer screening<br>• Breast cancer screening<br>• Cervical cancer screening<br>• Diabetes	• Blood pressure<br>• Diabetes
**Point of care (Completed)**	• Home blood pressure<br>• Suicide risk screening	• Home blood pressure<br>• Diabetes foot exam (modified video exam or reported)
**Orders (Planned)**	• Influenza vaccine<br>• Colorectal cancer screening<br>• Breast cancer screening<br>• Cervical cancer screening (visit scheduled)	• Hemoglobin A1c<br>• Microalbumin<br>• Diabetes foot exam (visit scheduled)<br>• Diabetic retinopathy screening/ monitoring (visit scheduled)

© 2025 Wheat CL et al. *JAMA Network Open*.

eTable 2. Chronic Disease Management Measures (eQMs) and Potentially Preventable Utilization Measures

Measure	*Description*	*Numerator*	*Denominator*	*Preferred Score Direction*
**Chronic Disease Measures**				
HbA1c poor control (dmg23h_ec)	Proportion of Veterans with diabetes not in control	Veterans with most recent HbA1c greater than 9 or no evidence of test within measurement year	Veterans 18-75 years old with a diagnosis of DM	Lower is better
Statin therapy for patients with diabetes (statn7_ec)	Proportion of Veterans with diabetes prescribed statins	Veterans with at least one dispensing of statin of any intensity within measurement year	Veterans 40-75 years old with a diagnosis of DM	Higher is better
Controlling High Blood Pressure (ihd53h_ec)	Proportion of Veterans with control of blood pressure	Veterans with most recent recorded BP as < 140/90mmHg	Veterans 18-85 years old with a diagnosis of hypertension	Higher is better
**Potentially Preventable Utilization Measures**				
Preventable ED visits	New York University (NYU) ED visit algorithm to assess the probability that ED visits are preventable			Lower is better
Ambulatory care sensitive hospitalizations	Admissions for acute Prevention Quality Indicator conditions			Lower is better

Abbreviations: HbA1c = Hemoglobin A1c; BP = Blood pressure; DM = Diabetes Mellitus; ED = Emergency department

© 2025 Wheat CL et al. *JAMA Network Open*.

eTable 3. Propensity Match Variable Pre and Post Match, HbA_{1c} Poor Control (dmg23h_ec)

	Pre-propensity Match			Post-propensity Match		
	Non-PHI recipients (n = 359,875) (M/SD or N/%)	PHI recipients (n = 36,955) (M/SD or N/%)	Standardized Mean Difference (p-value)	Non-PHI recipients (n = 7,538) (M/SD or N/%)	PHI recipients (n = 11,468) (M/SD or N/%)	Standardized Mean Difference (p-value)
	**Veteran characteristics**					
**Female sex&**	21,146 (6.7)	2,695 (7.3)	0.02 (<0.001)	506 (6.7)	789 (6.9)	0.01 (0.7)
**Married&**	216,069 (69)	25,013 (68)	0.03 (<0.001)	5,128 (68)	7,739 (67)	0.01 (0.4)
**Race/Ethnicity#**			0.10 (<0.001)			0.01 (>0.9)
Non-Hispanic White	206,854 (70)	23,889 (69)		5,155 (68)	7,896 (69)	
Non-Hispanic Black	75,007 (25)	8,474 (24)		1,915 (25)	2,864 (25)	
Hispanic	5,470 (1.8)	1,118 (3.2)		236 (3.1)	347 (3.0)	
Asian/Pacific Islander/Native Hawaiian	6,764 (2.3)	957 (2.8)		184 (2.4)	287 (2.5)	
American Indian/Alaska Native	3,041 (1.0)	272 (0.8)		48 (0.6)	74 (0.6)	
**Priority Status&**			0.01 (0.13)			0.01 (0.8)
1-3	211,208 (67)	24,739 (67)		4,931 (65)	7,546 (66)	
4-6	61,521 (20)	7,412 (20)		1,535 (20)	2,325 (20)	
7-8	40,798 (13)	4,800 (36,951)		1,072 (14)	1,597 (14)	
**Rurality&**			0.25 (<0.001)			0.02 (0.3)
Urban	193,627 (62)	27,107 (73)		5,395 (72)	8,308 (72)	
Rural	106,269 (34)	8,924 (24)		1,910 (25)	2,843 (25)	
Highly Rural/Insular Islands	13,505 (4.3)	913 (2.5)		233 (3.1)	317 (2.8)	
**Drive Distance to PC (per 10 miles) &**	1.63 (1.49)	1.38 (1.29)	0.18 (<0.001)	1.47 (1.42)	1.43 (1.45)	0.03 (<0.001)
**Outpatient Utilization (quartile in visits) &**			0.13 (<0.001)			0.10 (<0.001)
[0,7]	86,230 (28)	9,007 (24)		1,641 (22)	2,196 (19)	
(7,14]	80,837 (26)	8,556 (23)		1,972 (26)	2,834 (25)	
(14,26]	73,589 (23)	8,787 (24)		2,011 (27)	3,514 (31)	
(26,915]	72,900 (23)	10,605 (29)		1,914 (25)	2,924 (25)	
**Care Assessment Needs score (CAN) &**	58.55 (24.34)	61.23 (23.94)	-0.11 (<0.001)	60.02 (24.02)	61.85 (23.81)	-0.08 (<0.001)
**Gagne Score@**	0.60 (1.44)	0.65 (1.47)	-0.04 (<0.001)	0.58 (1.39)	0.64 (1.48)	-0.05 (0.021)
**Socioeconomic status (decile) &**			0.06 (<0.001)			0.04 (0.6)
0	26,620 (8.6)	3,767 (10)		682 (9)	1,063 (9.3)	
1	32,591 (11)	3,792 (10)		754 (10)	1,136 (9.9)	
2	37,184 (12)	4,387 (12)		798 (11)	1,255 (11)	
3	38,523 (12)	4,354 (12)		838 (11)	1,244 (11)	
4	37,191 (12)	4,508 (12)		945 (13)	1,414 (12)	
5	35,855 (12)	4,203 (11)		882 (12)	1,372 (12)	
6	33,332 (11)	3,809 (10)		830 (11)	1,290 (11)	

© 2025 Wheat CL et al. *JAMA Network Open*.

7	30,254 (9.8)	3,399 (9.3)		735 (9.8)	1,098 (9.6)	
8	24,361 (7.9)	2,835 (7.7)		625 (8.3)	995 (8.7)	
9	13,641 (4.4)	1,593 (4.3)		449 (6)	601 (5.2)	
	**Provider and Site Characteristics**					
**PCP VA Tenure (years)** [*]	14.25 (8.29)	14.20 (8.12)	0.01 (0.5)	14.51 (8.23)	14.38 (8.34)	0.02 (0.15)
**PHI Use (quartile)** [&]			1.8 (<0.001)			0.20 (<0.001)
[0,411]	87,813 (28)	277 (0.8)		65 (0.9)	46 (0.4)	
(411,1550]	85,489 (27)	849 (36,807)		248 (3.3)	227 (2.0)	
(1550,8680]	81,328 (26)	5,486 (15)		1,889 (25)	2,136 (19)	
(8680,623000]	56,569 (18)	30,195 (82)		5,336 (71)	9,059 (79)	
**Staffing Ratio (quartile)** [&]			0.08 (<0.001)			0.03 (0.14)
[0,2.79]	77,730 (25)	9,379 (25)		1,891 (25)	2,806 (24)	
(2.79,3.12]	76,889 (25)	10,162 (28)		2,183 (29)	3,354 (29)	
(3.12,3.68]	78,330 (25)	8,686 (24)		1,702 (23)	2,731 (24)	
(3.68,46.4]	78,350 (25)	8,601 (23)		1,762 (23)	2,577 (22)	
**Panel fullness (quartile)***		0.12 (<0.001)				0.05 (0.004)
[0,0.697]	53,797 (25)	6,822 (26)		2,024 (27)	3,158 (28)	
(0.697,0.778]	53,445 (25)	6,559 (25)		1,852 (25)	3,021 (26)	
(0.778,0.865]	52,557 (25)	7,423 (28)		2,021 (27)	2,974 (26)	
(0.865,1.0]	54,611 (25)	5,575 (21)		1,641 (22)	2,315 (20)	

[&] 12-13% missing data, [#] 16% missing data, [@] 19% missing data, * 39% missing data in pre-propensity matched sample, post-propensity matched complete case analysis

© 2025 Wheat CL et al. *JAMA Network Open*.

eTable 4. Propensity Match Variable Pre and Post Match, Statin Therapy for Patients With Diabetes (statn7_ec)

	Pre-propensity Match			Post-propensity Match		
	Non-PHI recipients (n = 265,607) (M/SD or N/%)	PHI recipients (n = 27,165) (M/SD or N/%)	Standardized Mean Difference (p-value)	Non-PHI recipients (n = 5,574) (M/SD or N/%)	PHI recipients (n = 8,434) (M/SD or N/%)	Standardized Mean Difference (p-value)
	**Veteran characteristics**					
**Female sex&**	16,872 (7.3)	2,125 (7.8)	0.06 (<0.001)	394 (7.1)	646 (92)	0.02 (0.2)
**Married&**	159,620 (69)	18,325 (67)	0.03 (<0.001)	3,780 (68)	5,661 (67)	0.01 (0.4)
**Race/Ethnicity#**			0.10 (<0.001)			0.02 (0.9)
Non-Hispanic White	148,745 (68)	17,013 (67)		3,712 (67)	5,614 (67)	
Non-Hispanic Black	59,777 (27)	6,742 (26)		1,536 (28)	2,295 (27)	
Hispanic	4,180 (1.9)	848 (3.3)		158 (2.8)	262 (3.1)	
Asian/Pacific Islander/Native Hawaiian	5,079 (2.3)	692 (2.7)		135 (2.4)	208 (2.5)	
American Indian/Alaska Native	2,203 (1.0)	201 (0.8)		33 (0.6)	55 (0.7)	
**Priority Status&**			0.01 (0.4)			0.01 (0.9)
1-3	155,620 (67)	18,103 (67)		3,664 (66)	5,513 (65)	
4-6	45,245 (19)	5,382 (20)		1,107 (20)	1,687 (20)	
7-8	31,410 (14)	3,678 (14)		803 (14)	1,234 (15)	
**Rurality&**			0.25 (<0.001)			0.02 (0.5)
Urban	144,976 (62)	20,023 (74)		4,012 (72)	6,140 (73)	
Rural	77,286 (33)	6,462 (24)		1,394 (25)	2,053 (24)	
Highly Rural/Insular Islands	9,924 (4.3)	670 (2.5)		168 (3.0)	241 (2.9)	
**Drive Distance to PC (per 10 miles) &**	1.62 (1.49)	1.37 (1.29)	0.18 (<0.001)	1.44 (1.33)	1.42 (1.47)	0.02 (0.003)
**Outpatient Utilization (quartile in visits) &**			0.14 (<0.001)			0.11 (<0.001)
[0,6]	68,316 (29)	6,999 (26)		1,349 (24)	1,756 (21)	
(6,12]	55,425 (24)	5,829 (21)		1,316 (24)	1,924 (23)	
(13,23]	55,152 (24)	6,531 (24)		1,422 (26)	2,138 (25)	
(23,702]	53,406 (23)	7,806 (29)		1,487 (27)	2,616 (31)	
**Care Assessment Needs score (CAN) &**	56.00 (24.17)	58.69 (23.83)	-0.11 (<0.001)	57.61 (24.15)	59.26 (23.77)	-0.07 (<0.001)
**Gagne Score@**	0.44 (1.29)	0.48 (1.30)	-0.03 (<0.001)	0.46 (1.31)	0.48 (1.30)	-0.01 (0.3)
**Socioeconomic status (decile) &**			0.07 (<0.001)			0.04 (0.7)
0	20,337 (8.9)	2,938 (11)		580 (10)	819 (9.7)	
1	24,466 (11)	2,841 (11)		566 (10)	858 (10)	
2	27,518 (12)	3,197 (12)		634 (11)	912 (11)	
3	28,411 (12)	3,165 (12)		616 (11)	910 (11)	
4	27,381 (12)	3,250 (12)		639 (11)	1,021 (12)	
5	26,232 (11)	3,075 (11)		648 (12)	1,022 (12)	

© 2025 Wheat CL et al. *JAMA Network Open*.

6	24,458 (11)	2,754 (10)		571 (10)	925 (11)	
7	22,253 (9.7)	2,481 (9.2)		543 (9.7)	807 (9.6)	
8	18,118 (7.9)	2,079 (7.7)		481 (8.6)	730 (8.7)	
9	10,172 (4.4)	1,164 (4.3)		296 (5.3)	430 (5.1)	
	**Provider and Site Characteristics**					
**PCP VA Tenure (years)***	14.24 (8.28)	14.22 (8.07)	0.00 (0.2)	14.40 (8.19)	14.28 (8.27)	0.02 (0.2)
**PHI Use (quartile) &**			1.8 (<0.001)			0.19 (<0.001)
[0,418]	64,712 (28)	203 (0.8)		61 (1.1)	35 (0.4)	
(418,1530]	63,696 (28)	616 (2.3)		152 (2.7)	159 (1.9)	
(1530,8230]	60,107 (26)	4,046 (15)		1,378 (25)	1,554 (18)	
(8230,623000]	42,003 (18)	22,199 (82)		3,983 (71)	6,686 (79)	
**Staffing Ratio (quartile) &**						0.04 (0.2)
[0,2.74]	57,574 (25)	6,869 (25)	0.07 (<0.001)	1,394 (25)	2,030 (24)	
(2.74,3.11]	57,117 (25)	7,373 (27)		1,547 (28)	2,471 (29)	
(3.11,3.72]	57,781 (25)	6,543 (24)		1,394 (25)	2,117 (25)	
(3.72,46.4]	58,105 (25)	6,293 (23)		1,239 (22)	1,816 (22)	
**Panel fullness (quartile)***			0.13 (<0.001)			0.03 (0.3)
[0,0.699]	39,832 (25)	5,103 (26)		1,486 (27)	2,336 (28)	
(0.699,0.78]	39,596 (25)	4,848 (25)		1,436 (26)	2,207 (26)	
(0.78,0.866]	39,085 (25)	5,540 (28)		1,505 (27)	2,258 (27)	
(0.866,1.0]	40,570 (26)	4,039 (21)		1,147 (21)	1,633 (19)	

& 11-12% missing data, # 16% missing data, @ 19% missing data, * 39% missing data in pre-propensity matched sample, post-propensity matched complete case analysis

© 2025 Wheat CL et al. *JAMA Network Open*.

eTable 5. Propensity Match Variable Pre and Post Match, Controlling High Blood Pressure (ihd53h_ec)

	Pre-propensity Match			Post-propensity Match		
	Non-PHI recipients (n = 520,187) (M/SD or N/%)	PHI recipients (n = 60,087) (M/SD or N/%)	Standardized Mean Difference (p-value)	Non-PHI recipients (n = 10,738) (M/SD or N/%)	PHI recipients (n = 17,433) (M/SD or N/%)	Standardized Mean Difference (p-value)
	**Veteran characteristics**					
**Female sex&**	29,022 (6.3)	4,025 (6.7)	0.02 (<0.001)	643 (6.0)	1,026 (5.9)	0.00 (0.7)
**Married&**	318,589 (69)	40,546 (67)	0.03 (<0.001)	7,318 (68)	11,765 (67)	0.01 (0.2)
**Race/Ethnicity#**			0.08 (<0.001)			0.02 (0.5)
Non-Hispanic White	318,531 (72)	41,073 (72)		7,650 (71)	12,547 (72)	
Non-Hispanic Black	105,688 (24)	12,950 (23)		2,577 (24)	4,068 (23)	
Hispanic	5,789 (1.3)	1,263 (2.2)		229 (2.1)	398 (2.3)	
Asian/Pacific Islander/Native Hawaiian	7,143 (1.6)	1,087 (1.9)		216 (2.0)	323 (1.9)	
American Indian/Alaska Native	3,473 (0.8)	334 (0.6)		66 (0.6)	97 (0.6)	
**Priority Status&**			0.01 (0.3)			0.02 (0.3)
1-3	295,605 (64)	38,300 (64)		6,744 (63)	10,990 (63)	
4-6	101,573 (22)	13,277 (22)		2,363 (22)	3,905 (22)	
7-8	66,570 (14)	8,508 (14)		1,631 (15)	2,538 (15)	
**Rurality&**			0.31 (<0.001)			0.06 (<0.001)
Urban	278,929 (60)	44,667 (74)		7,778 (72)	13,099 (75)	
Rural	163,628 (35)	14,072 (23)		2,671 (25)	3,924 (23)	
Highly Rural/Insular Islands	21,017 (4.5)	1,331 (2.2)		289 (2.7)	410 (2.4)	
**Drive Distance to PC (per 10 miles) &**	1.63 (1.48)	1.35 (1.24)	0.21 (<0.001)	1.41 (1.30)	1.34 (1.37)	0.05 (<0.001)
**Outpatient Utilization (quartile in visits) &**			0.13 (<0.001)			0.11 (<0.001)
[0,6]	128,610 (28)	14,459 (24)		2,312 (22)	3,177 (18)	
(6,13]	119,090 (26)	14,116 (23)		2,788 (26)	4,265 (24)	
(13,24]	103,268 (22)	13,782 (23)		2,506 (23)	4,183 (24)	
(24,932]	112,808 (24)	17,730 (30)		3,132 (29)	5,808 (33)	
**Care Assessment Needs score (CAN) &**	57.11 (25.54)	59.87 (24.97)	-0.11 (<0.001)	59.18 (25.12)	61.48 (24.63)	-0.09 (<0.001)
**Gagne Score@**	0.16 (1.36)	0.23 (1.39)	-0.05 (<0.001)	0.16 (1.35)	0.23 (1.40)	-0.05 (<0.001)
**Socioeconomic status (decile) &**			0.06 (<0.001)			0.04 (0.4)
0	38,034 (8.3)	5,811 (9.8)		964 (9.0)	1,557 (8.9)	
1	46,533 (10)	5,749 (9.7)		1,041 (9.7)	1,677 (9.6)	
2	53,828 (12)	6,778 (11)		1,204 (11)	1,849 (11)	
3	56,704 (12)	7,084 (12)		1,214 (11)	1,900 (11)	
4	55,220 (12)	7,419 (12)		1,370 (13)	2,143 (12)	
5	53,211 (12)	6,771 (11)		1,249 (12)	2,038 (12)	
6	49,231 (11)	6,217 (10)		1,150 (11)	1,897 (11)	
7	45,463 (9.9)	5,821 (9.8)		1,015 (9.5)	1,752 (10)	

© 2025 Wheat CL et al. *JAMA Network Open*.

8	37,825 (8.3)	5,066 (8.5)		945 (8.8)	1,622 (9.3)	
9	21,753 (4.8)	2,854 (4.8)		586 (5.5)	998 (5.7)	
	**Provider and Site Characteristics**					
**PCP VA Tenure (years)** *	14.42 (8.36)	14.27 (8.01)	0.02 (>0.9)	14.52 (8.19)	14.45 (8.16)	0.01 (0.5)
**PHI Use (quartile)** &			1.9 (<0.001)			0.22 (<0.001)
[0,427]	130,934 (28)	407 (0.7)		71 (0.7)	50 (0.3)	
(427,1590]	128,422 (28)	1,191 (2.0)		275 (2.6)	277 (1.6)	
(1590,9920]	121,723 (26)	8,161 (14)		2,588 (24)	2,870 (16)	
(9920,623000]	79,771 (17)	50,191 (84)		7,804 (73)	14,236 (82)	
**Staffing Ratio (quartile)** &			0.17 (<0.001)			0.07 (<0.001)
[0,2.76]	114,377 (25)	16,162 (27)		2,736 (25)	4,282 (25)	
(2.76,3.11]	112,261 (24)	18,054 (30)		3,282 (31)	5,761 (33)	
(3.11,3.72]	116,438 (25)	13,573 (23)		2,408 (22)	3,996 (23)	
(3.72,46.4]	118,042 (26)	12,161 (20)		2,312 (25)	3,394 (19)	
**Panel fullness (quartile)***			0.10 (<0.001)			0.06 (<0.001)
[0,0.698]	80,690 (25)	10,097 (23)		2,669 (25)	4,467 (26)	
(0.698,0.777]	80,899 (25)	10,727 (25)		2,705 (25)	4,689 (27)	
(0.777,0.861]	77,619 (24)	12,312 (29)		2,931 (23)	4,703 (27)	
(0.861,1.0]	80,569 (25)	9,947 (23)		2,433 (25)	3,574 (21)	

[&] 10-11% missing data, [#] 14% missing data, [@] 17% missing data, * 36-37% missing data in pre-propensity matched sample, post-propensity matched complete case analysis

© 2025 Wheat CL et al. *JAMA Network Open*.

eTable 6. Propensity Match Variable Pre and Post Match, Utilization Outcomes (Preventable ED Visits, Ambulatory Care Sensitive Hospitalizations, Outpatient VA Utilization)

	Pre-propensity Match			Post-propensity Match		
	Non-PHI recipients (n = 3,932,579) (M/SD or N/%)	PHI recipients (n = 409,056) (M/SD or N/%)	Standardized Mean Difference (p-value)	Non-PHI recipients (n = 118,188) (M/SD or N/%)	PHI recipients (n = 97,695) (M/SD or N/%)	Standardized Mean Difference (p-value)
	**Veteran characteristics**					
**Female sex&**	406,953 (10)	42,221 (10)	0.00 (0.6)	10,498 (8.9)	8,371 (8.6)	0.01 (0.10)
**Married&**	2,755,921 (70)	280,374 (69)	0.03 (<0.001)	81,467 (69)	66,391 (68)	0.02 (<0.001)
**Race/Ethnicity#**			0.08 (<0.001)			0.02 (<0.001)
Non-Hispanic White	2,767,177 (76)	287,416 (76)		87,994 (74)	73,557 (75)	
Non-Hispanic Black	708,912 (19)	69,793 (18)		23,700 (20)	18,882 (19)	
Hispanic	65,608 (1.8)	11,107 (2.9)		2,903 (2.5)	2,483 (2.5)	
Asian/Pacific Islander/Native Hawaiian	80,779 (2.2)	9,076 (2.4)		2,708 (2.3)	2,094 (2.1)	
American Indian/Alaska Native	34,232 (0.9)	2,586 (0.7)		880 (0.7)	674 (0.7)	
**Priority Status&**			0.03 (<0.001)			0.01 (0.3)
1-3	2,473,600 (63)	252,982 (62)		72,377 (61)	59,582 (61)	
4-6	834,968 (21)	90,854 (22)		26,479 (22)	22,164 (23)	
7-8	603,112 (15)	63,332 (16)		19,329 (16)	15,944 (16)	
**Rurality&**			0.28 (<0.001)			0.05 (<0.001)
Urban	2,518,317 (64)	313,379 (77)		86,262 (73)	73,340 (75)	
Rural	1,231,568 (31)	85,156 (21)		28,881 (24)	22,013 (23)	
Highly Rural/Insular Islands	160,070 (4.1)	8,521 (2.1)		3,042 (2.6)	2,337 (2.4)	
**Drive Distance to PC (per 10 miles) &**	1.57 (1.46)	1.32 (1.23)	0.19 (<0.001)	1.42 (1.31)	1.36 (1.37)	0.04 (<0.001)
**Outpatient Utilization (quartile in visits) &**			0.17 (<0.001)			0.12 (<0.001)
[0,3]	1,115,066 (28)	95,581 (23)		18,313 (15)	12,766 (13)	
(3,8]	965,209 (25)	89,253 (22)		28,360 (24)	21,219 (22)	
(8,18]	913,894 (23)	98,822 (24)		32,236 (27)	26,432 (27)	
(18,1001]	938,410 (24)	125,400 (31)		39,276 (33)	37,273 (38)	
**Care Assessment Needs score (CAN) &**	47.09 (28.58)	51.65 (28.57)	-0.16 (<0.001)	55.05 (26.63)	57.78 (26.82)	-0.10 (<0.001)
**Gagne Score***	0.51 (1.56)	0.60 (1.63)	-0.06 (<0.001)	0.51 (1.51)	0.58 (1.61)	-0.05 (<0.001)
**Socioeconomic status (decile) &**			0.09 (<0.001)			0.01 (0.7)
0	255,919 (6.6)	36,104 (8.9)		9,622 (8.1)	7,932 (8.1)	

© 2025 Wheat CL et al. *JAMA Network Open*.

1	337,809 (8.7)	35,781 (8.8)		10,424 (8.8)	8,568 (8.8)	
2	398,367 (10)	41,777 (10)		11,770 (10)	9,723 (10)	
3	441,007 (11)	44,305 (11)		12,513 (11)	10,153 (10)	
4	448,107 (12)	48,595 (12)		13,943 (12)	11,793 (12)	
5	459,505 (12)	46,653 (12)		13,954 (12)	11,528 (12)	
6	449,577 (12)	44,947 (11)		13,352 (11)	11,003 (11)	
7	439,003 (11)	43,258 (11)		12,830 (11)	10,545 (11)	
8	380,028 (9.8)	39,262 (9.7)		11,880 (10)	9,927 (10)	
9	251,910 (6.5)	23,802 (5.9)		7,897 (6.7)	6,518 (6.7)	
	**Provider and Site Characteristics**					
**PCP VA Tenure (years)** *	13.99 (8.22)	13.97 (8.09)	0.00 (<0.001)	14.15 (8.14)	14.09 (8.19)	0.01 (0.033)
**PHI Use (quartile)** &			1.9 (<0.001)			0.22 (<0.001)
[0,411]	1,076,949 (28)	3,153 (0.8)		902 (0.8)	323 (0.3)	
(411,1560]	1,081,127 (28)	9,208 (2.3)		3,334 (2.8)	1,640 (1.7)	
(1560,8510]	1,013,158 (26)	51,987 (13)		26,910 (23)	15,093 (15)	
(8510,623000]	731,560 (19)	343,496 (84)		87,039 (74)	80,634 (83)	
**Staffing Ratio (quartile)** &			0.16 (<0.001)			0.05 (<0.001)
[0,2.75]	970,931 (25)	117,417 (29)		30,662 (26)	25,143 (26)	
(2.75,3.09]	952,728 (24)	114,595 (28)		34,052 (29)	29,784 (30)	
(3.09,3.68]	987,977 (25)	92,855 (23)		27,762 (23)	23,315 (24)	
(3.68,46.4]	991,687 (25)	83,065 (20)		25,709 (22)	19,448 (20)	
**Panel fullness (quartile)***			0.09 (<0.001)			0.05 (<0.001)
[0,0.698]	665,488 (25)	70,409 (25)		31,309 (26)	26,428 (27)	
(0.698,0.777]	650,128 (25)	73,632 (26)		29,723 (25)	25,831 (26)	
(0.777,0.861]	653,999 (25)	79,150 (28)		32,113 (27)	26,335 (27)	
(0.861,1.0]	664,105 (25)	62,326 (22)		25,040 (21)	19,096 (20)	

& <1% missing data, # 7% missing data, * 28-35% missing data in pre-propensity matched sample, post-propensity matched complete case analysis

© 2025 Wheat CL et al. *JAMA Network Open*.

eFigure 1. Monthly eQM Trends of Diabetes Control Pre and Post PHI Introduction: PHI Users vs Non–PHI Users

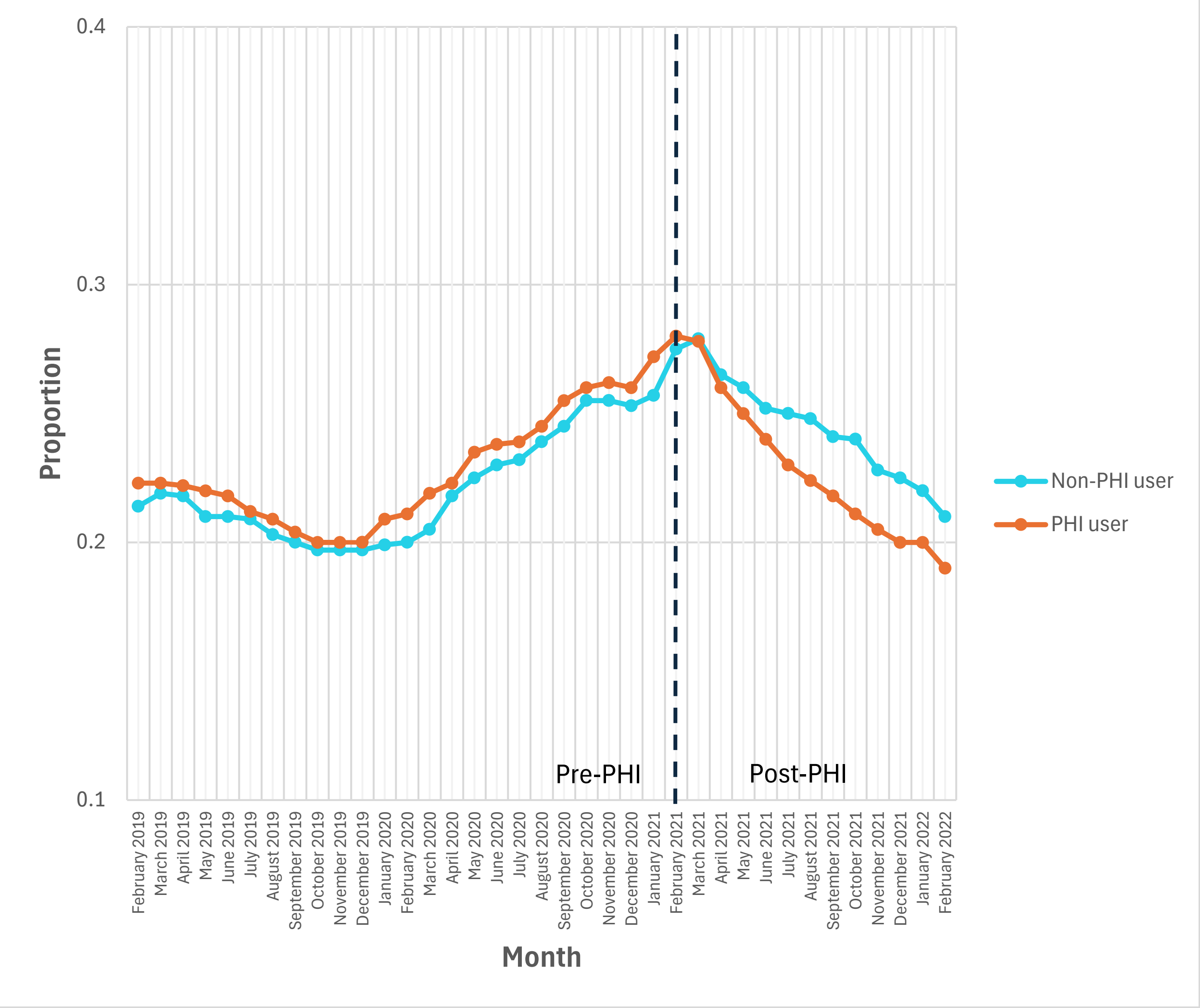

© 2025 Wheat CL et al. *JAMA Network Open*.

eFigure 2. Monthly eQM Trends of Statin Use in Diabetes Pre and Post PHI Introduction: PHI Users vs Non–PHI Users

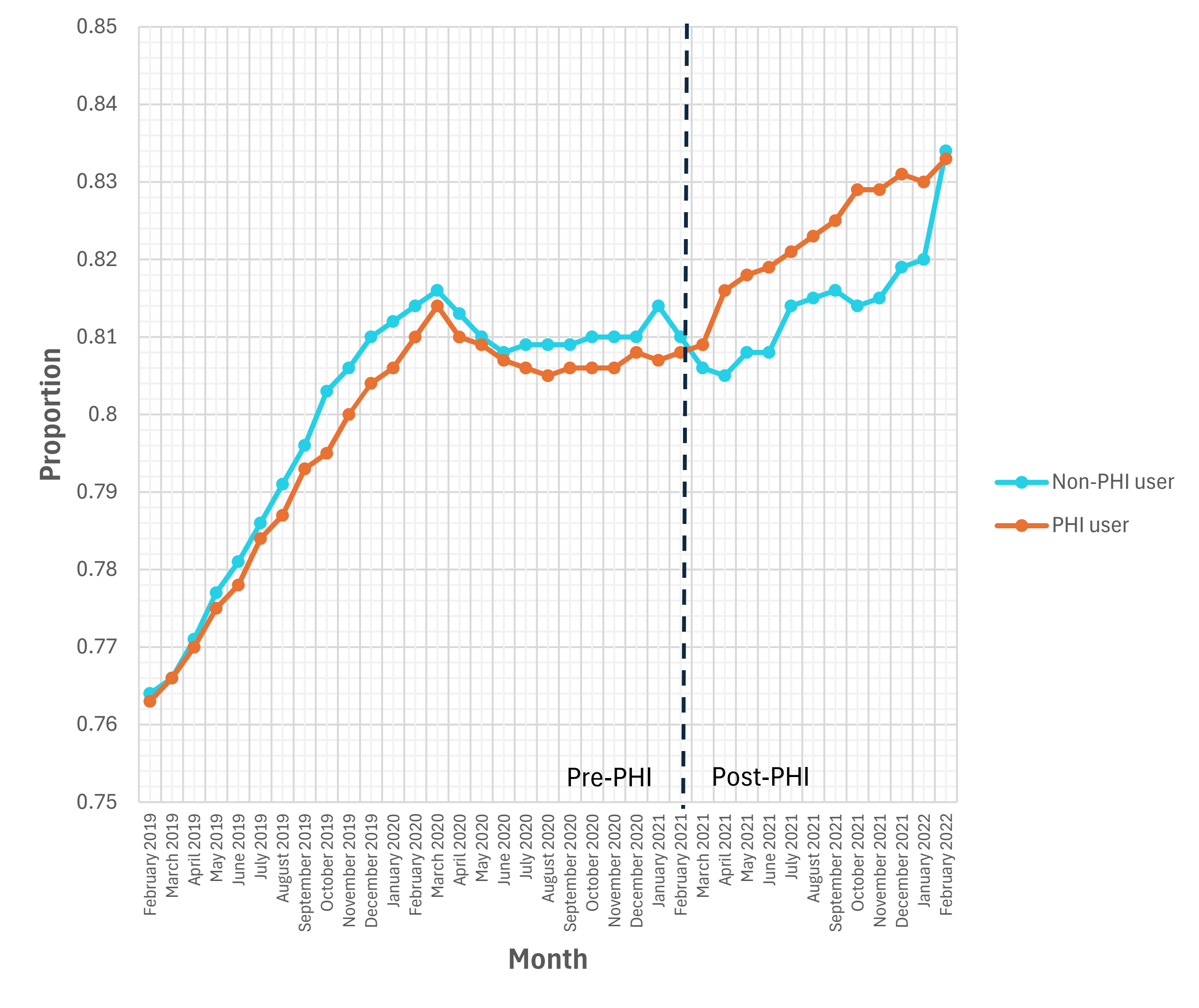

© 2025 Wheat CL et al. *JAMA Network Open*.

eFigure 3. Monthly eQM Trends of Blood Pressure Control for Hypertension Pre and Post PHI Introduction: PHI Users vs Non–PHI Users

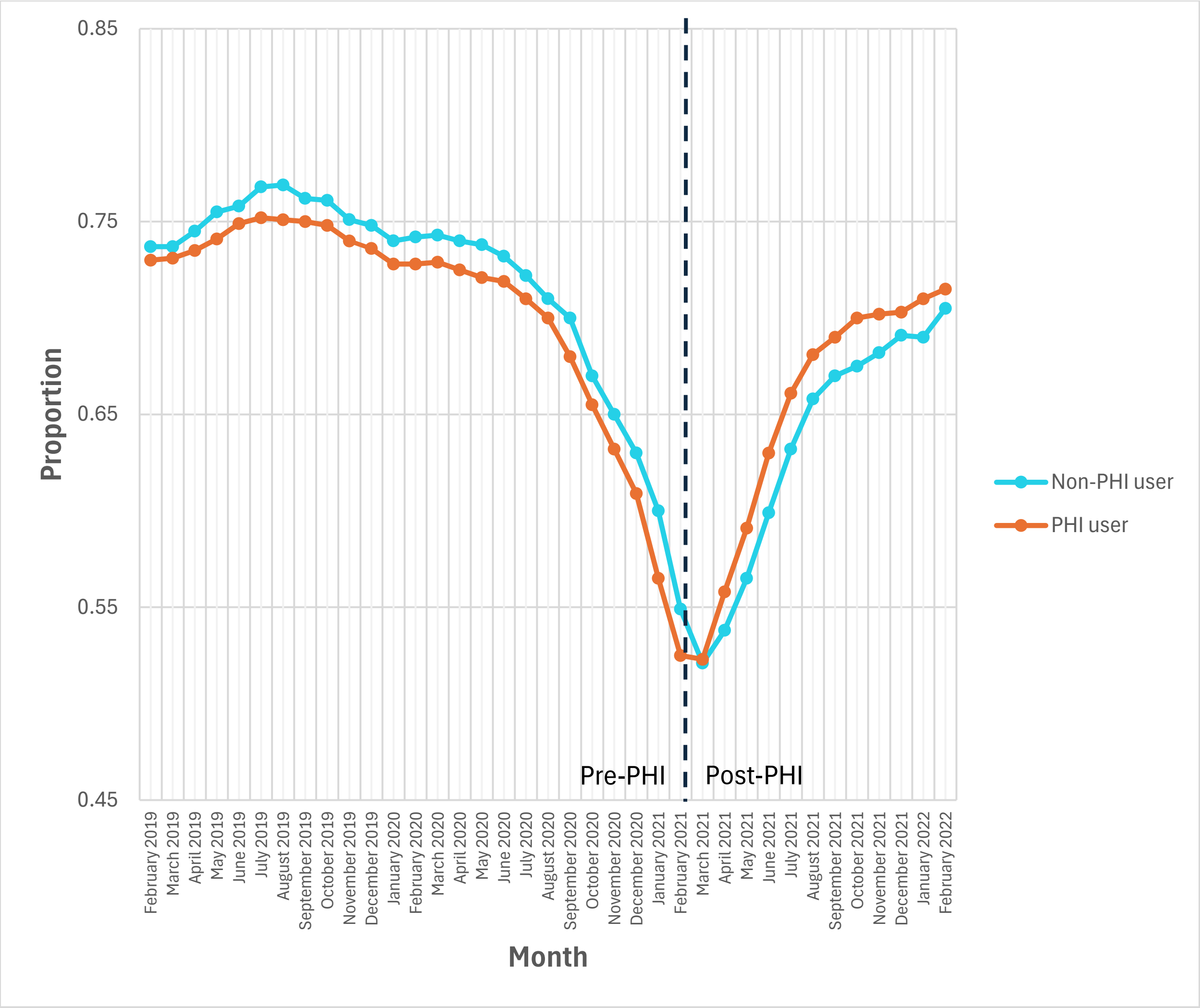

© 2025 Wheat CL et al. *JAMA Network Open*.

eFigure 4. Monthly eQM Trends of Ambulatory Care Sensitive Condition Hospitalizations Pre and Post PHI Introduction: PHI Users vs Non–PHI Users

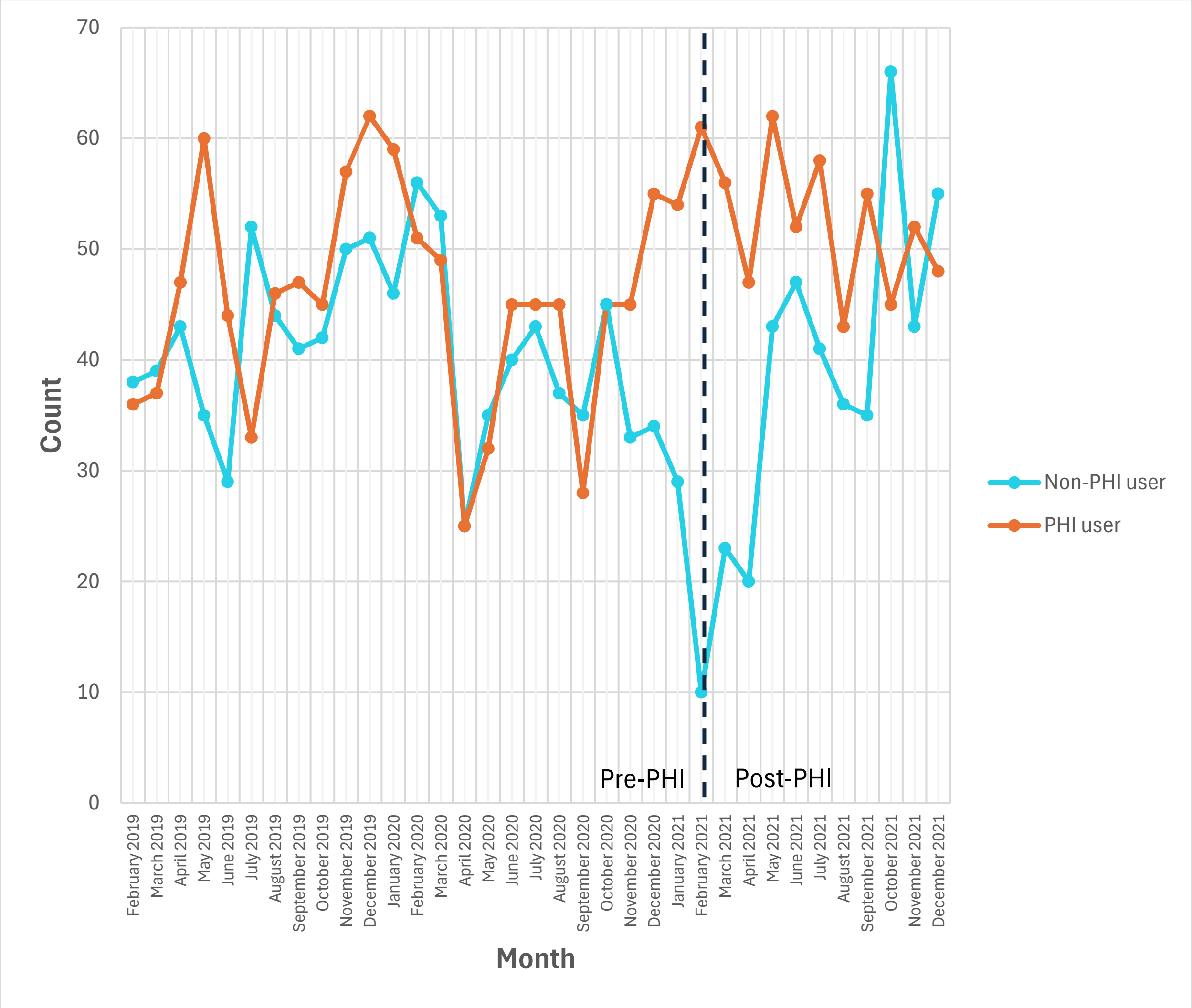

© 2025 Wheat CL et al. *JAMA Network Open*.

eFigure 5. Monthly eQM Trends of Preventable Emergency Department Visits Pre and Post PHI Introduction: PHI Users vs Non–PHI Users

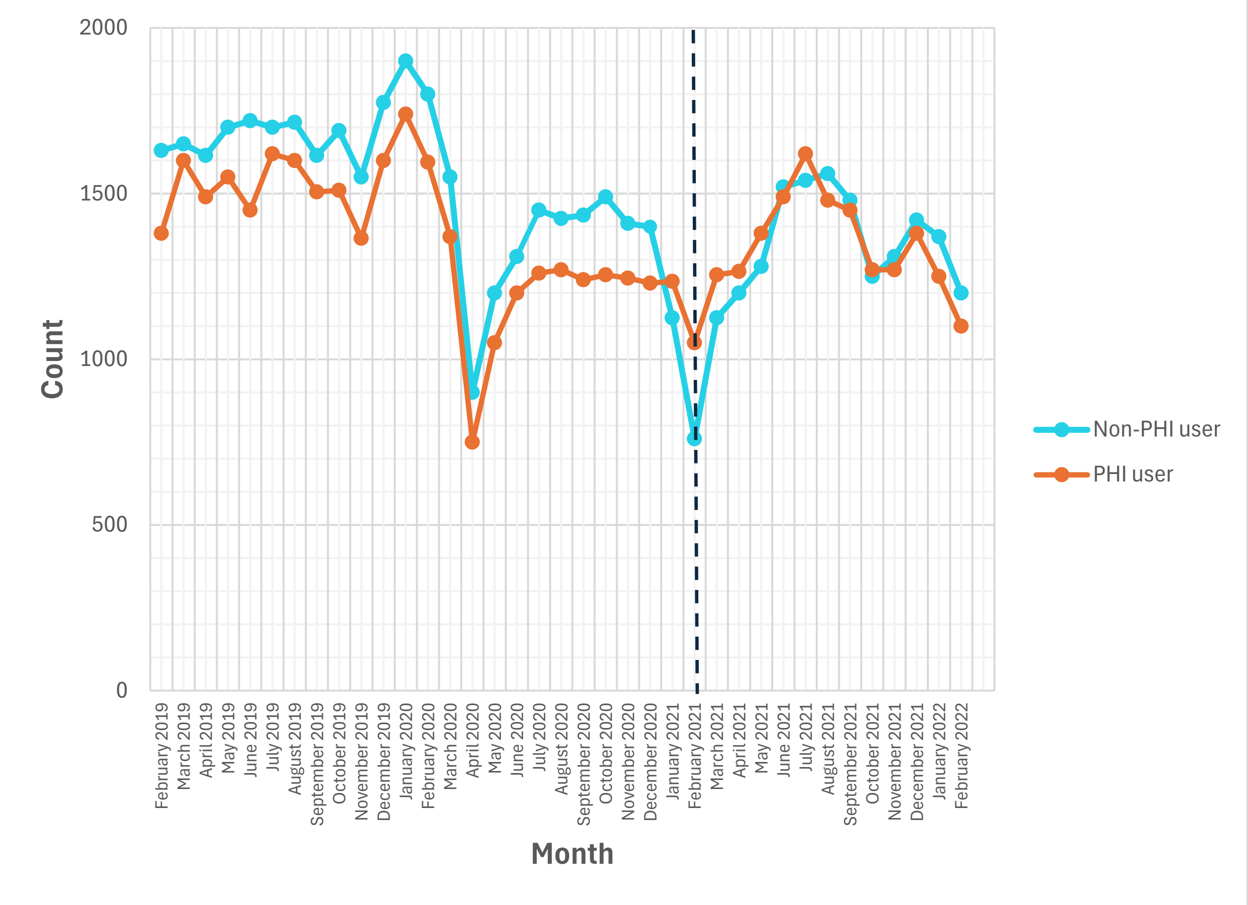

© 2025 Wheat CL et al. *JAMA Network Open*.

eFigure 6: Monthly eQM Trends of VA Outpatient Utilization Pre and Post PHI Introduction: PHI Users vs Non–PHI Users

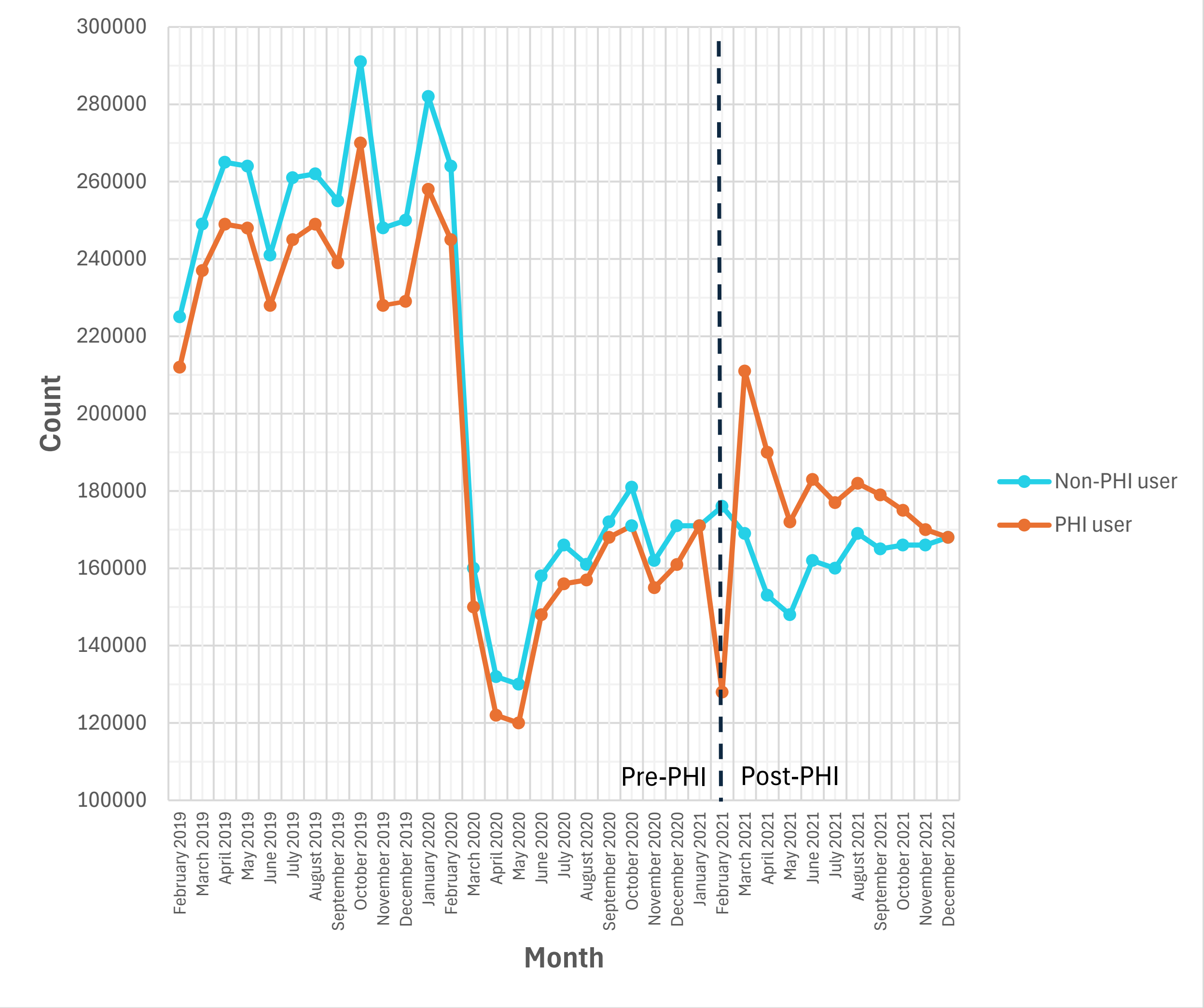

© 2025 Wheat CL et al. *JAMA Network Open*.
